# Milk Lacking α-Casein Leads to Permanent Reduction in Body Size in Mice

**DOI:** 10.1371/journal.pone.0021775

**Published:** 2011-07-18

**Authors:** Andreas F. Kolb, Reinhard C. Huber, Simon G. Lillico, Ailsa Carlisle, Claire J. Robinson, Claire Neil, Linda Petrie, Dorte B. Sorensen, I. Anna S. Olsson, C. Bruce A. Whitelaw

**Affiliations:** 1 Molecular Recognition Group, Hannah Research Institute, Ayr, United Kingdom; 2 Nutrition and Epigenetics Group, Vascular Health Division, Rowett Institute of Nutrition and Health, University of Aberdeen, Aberdeen, United Kingdom; 3 Laboratory Animal Science Group, Instituto de Biologia Molecular e Celular, Universidade do Porto, Porto, Portugal; 4 Division of Developmental Biology, The Roslin Institute and Royal (Dick) School of Veterinary Studies, University of Edinburgh, Edinburgh, United Kingdom; 5 Department of Large Animal Sciences, University of Copenhagen, Copenhagen, Denmark; 6 Danish Centre for Bioethics and Risk Assessment, Faculty of Life Sciences, University of Copenhagen, Copenhagen, Denmark; University of Frankfurt, Germany

## Abstract

The major physiological function of milk is the transport of amino acids, carbohydrates, lipids and minerals to mammalian offspring. Caseins, the major milk proteins, are secreted in the form of a micelle consisting of protein and calcium-phosphate.

We have analysed the role of the milk protein α-casein by inactivating the corresponding gene in mice. Absence of α-casein protein significantly curtails secretion of other milk proteins and calcium-phosphate, suggesting a role for α-casein in the establishment of casein micelles. In contrast, secretion of albumin, which is not synthesized in the mammary epithelium, into milk is not reduced. The absence of α-casein also significantly inhibits transcription of the other casein genes. α-Casein deficiency severely delays pup growth during lactation and results in a life-long body size reduction compared to control animals, but has only transient effects on physical and behavioural development of the pups. The data support a critical role for α-casein in casein micelle assembly. The results also confirm lactation as a critical window of metabolic programming and suggest milk protein concentration as a decisive factor in determining adult body weight.

## Introduction

Milk is a hallmark of mammals, providing the primary source of nutrition for their young until weaning. It is an emulsion of fat globules in a water-based fluid. The major proteins of this fluid are the caseins. The caseins are serine rich phosphoproteins which are almost exclusively expressed in the lactating mammary gland [1], [2]. In cows the casein proteins together constitute up to 80% of total milk protein and also 80% of total mRNA in the lactating mammary gland [3]. The caseins are members of a large family of serine rich phospho-proteins, which are clustered on chromosome 5 in the mouse (chromosome 6 in cattle, chromosome 4 in humans). There are 5 functional casein genes in the mouse whereas most other species (including ruminants) only express 3 or 4 functional genes [1]. The casein proteins show little homology with each other outside their signal peptide domain [2], [4].

Inactivation of the β-casein gene has shown to have little effect on milk secretion and growth of the offspring in the mouse [5]. In contrast, deletion of the κ-casein gene in mice completely abrogates milk production [6]. Exactly why this happens is unclear as neither the structure of the individual casein proteins nor the structure of the casein micelles is currently known in detail [7], [8], [9]. The available results, however, support a model in which κ-casein (the casein protein which is present in milk in least abundance) plays a critical role in ensuring transport and solubility of casein micelles [7]. The sequences of α- (αS1 in ruminants) β-, and γ-casein (αS2-casein in ruminants) contain one or more clusters of phosphorylated residues known as phosphate centres. Phosphate centres can sequester amorphous calcium phosphate, probably in the Golgi vesicles of mammary secretory cells, to form thermodynamically stable complexes of defined chemical composition which are then secreted through the apical membrane of the mammary epithelial cells [10], [11], [12], [13], [14]. Milk, like many other biological fluids, is supersaturated with respect to the crystalline mineral of bones and teeth (apatite) but due to the sequestration reaction, is under-saturated with respect to amorphous calcium phosphate. Since apatite only forms by maturation of the amorphous phase of calcium phosphate, it cannot form at all from milk and this helps to protect the mammary gland against soft tissue mineralization [15]. In most milks, the majority of the total calcium is sequestered within the casein micelles. In milks of different species, the total calcium and total casein concentrations are highly correlated [16] and provided this balance is maintained, the milks remain stable. If there is insufficient casein to sequester the secreted calcium and orthophosphate then the milk becomes unstable [15]. In mouse milk, 3 of the phosphate centres are in present α-casein and only one in β-casein. Thus the loss of α-casein is likely to have more serious consequences for milk stability than the loss of β-casein.

Deficiencies for β-casein and α-casein exist as naturally occurring genotypes in goats [17] but have no apparent detrimental effect on milk production. However, the αS1-Cn0 allele has been reported to decrease the efficiency with which other casein proteins are secreted [18]. We set out to define the role of α-casein in milk secretion and its nutritional role by inactivating the corresponding gene in mice. Our results demonstrate that α-casein deficiency has a significant effect on milk protein secretion and growth of the offspring.

## Materials and Methods

### DNA

PCR amplifications were done using Taq Polymerase from various suppliers. Oligonucleotides were purchased from MWG, Sigma-Genosys or Invitrogen. Primer sequences, amplicon size and annealing temperatures are given in table 1. Template DNA for PCR analyses was isolated as described [19].

**Table 1:** Primer combinations used for PCR analysis.

*name*	*sequence*	*annealing temp.*	*amplicon size*
acas6	5′ GTT CCA GTA CGC TAA GAG CTT AAA ACT GC 3′		
acas7	5′ TAG GGG GCA AAA ATG TGT ATT ATC CTA GC 3′	53°C	1566 bp
acas6	5′ GTT CCA GTA CGC TAA GAG CTT AAA ACT GC 3′		
pBKpA	5′ GCT ATT GCT TTA TTT GTA ACC ATT A 3′	53°C	848 bp
acas4	5′ AAG TTT CCC CAG CAC AGC AAT CT 3′		
acas5	5′ CCA AAG GGG AAA GGC ATC ATA CT 3′	56°C	248 bp
bcas21	5′ GAT GCC CCT CCT TAA CTC TGA AA 3′		
bcas22	5′ TTG TGG AAG GAA GGG TGC TAC T 3′	56°C	226 bp
gcas10	5′ ATA ACA CGC CCA CCC AGG AAT C 3′		
gcas11	5′ GGG AAA CCA CGA AGA AAC CAA T 3′	54°C	272 bp
kcas1	5′ TCG ACC CCA TTA CTC CCA TTG TGT 3′		
kcas2	5′ TGT AAA AGG TAA GGG AAG ACG AGA AAG AT 3′	53°C	289 bp
mGAPDH1	5′ GCT TTC CAG AGG GGC CAT CCA CA 3′		
mGAPDH2	5′ ACG GCA AAT TCA ACG GCA CAG TCA A 3′	61°C	426 bp
acas1	5′ ATG AAA CTC CTC ATC CTC ACC TGC 3′		
acas7	5′ TAG GGG GCA AAA ATG TGT ATT ATC CTA GC 3′	51°C	688 bp
PGK5	5′ AAG CGC ATG CTC CAG ACT GCC TTG GGA AA 3′		
acas7	5′ TAG GGG GCA AAA ATG TGT ATT ATC CTA GC 3′	52°C	450 bp
Nol3-1	5′ CTC CGG ACC ACA AGC CCG ACT C 3′		
Nol3-2	5′ CTG GGT GCT TCT GGC GTC CAG 3′	61°C	452 bp
Traf1-5	5′ GCA AAC CCT GGC TCA AAA AG 3′		
Traf1-6	5′ ACT CTG TGG CCG CTG GAA GG 3′	57°C	409 bp
Birc5-1	5 CTG GAG GAC TGC GCC TGC AC′ 3′		
Birc5-2	5′ TGC TAG GAG GCC CTG GCT GG 3′	65°C	432 bp
BiP-3	5′ TCA CGT CCA ACC CCG AGA ACA C 3′		
BiP-4	5′ GCC GCC ACC CAG GTC AAA CAC A 3′	58°C	434 bp
grp94-1	5′ GGG TGT TGG GCC TCT GCT GTG 3′		
grp94-2	5′ ACC CGT GTC TGT GAC ATG CAG C 3′	54°C	440 bp
PDIA6-1	5′ GGC AGC CAT CAA TGC ACG CAA 3′		
PDIA6-2	5′ TGG GCC GTG GCT CAC AAC TCA T 3′	58°C	258 bp
REDD1-1	5′ TGG TGC CCA CCT TTC AGT TG 3′		
REDD1-2	5′ GTC AGG GAC TGG CTG TAA CC 3′	53°C	121 bp

The targeting construct for the α-casein gene was generated using a short arm of homology of 679 bp corresponding to nucleotides 208 to 887 downstream of the transcriptional start site (which was isolated as a EcoRV/BssHII fragment from the bacterial artificial chromosome BAC 490H23 of a Research Genetics BeloBAC11 library derived from 129SV mouse DNA) and a long arm of homology of 6696 bp corresponding to nucleotides 1422 to 8117 downstream of the transcriptional start site (isolated as a StuI/EcoRV fragment). The targeting construct carries a hygromycin-thymidine kinase fusion gene under the control of the murine phospho-glycerol kinase (PGK) promoter as selection marker.

### Mouse generation

Transgenic mice were generated at Genoway (Lyon, France) as a SV129xC57BL/6 mixed background. After transfer to the transgenic mouse facility at the Roslin Institute the mice were maintained there in accordance with Home Office guidelines. This study was approved by the Roslin Institute Animal Ethics Committee and was performed under Home Office Licence 60/3779.

### Housing and maintenance of mice

Before establishing the mating couples, mice were kept in same-sex groups of 2 to 6 littermates. The animals were kept in opaque M3 type cages with dimensions of 48(15(13(cm from North Kent Plastics, UK equipped with bedding (‘Eco-bedding’, B&K Universal Ltd, UK) and nesting material (‘Nestlet’, Datesand Ltd, UK). Cages were changed once per week and in case of the nursing females the nesting material was transferred to the new cage. The environment was kept at 21+/(2(C and 55+/(15% relative air humidity with a 12(hour light 12(hour dark cycle (lights off 8:00 pm) Food (BeeKay Transgenic Rodent Diet BK021E, B&K Universal Ltd, UK) and tap water were available ad libitum, water bottles being routinely changed twice per week.

### Breeding of mice

Apparently pregnant females were separated from the male and checked daily in the morning for pups in the cage. The first day pups were found was considered the day of birth (day 1). On this day the pups were marked individually by foot pad tattooing and checked for the presence of a milk spot and signs of lack of maternal care. The offspring of 3(wt females were fostered onto 3 (-casein deficient females and vice versa on day 2 (Table 2), the cross fostered litters being matched for day of birth. The pups were weaned on day 23 by separating them from the nursing female and placing them in cages as described above with same-sex litter mates. From weaning until the age of 6 weeks, water-soaked diet was provided ad libitum in Petri dishes on the cage to weaned pups in all groups to facilitate solid food intake by growth impaired animals.

**Table 2:** Experimental groups.

*Group*	*Females*	*Pups nursed*	*n litters*	*n pups*	*n pups/litter min-max on day 21*
G1	Wt +/+	Wt +/+ (own)	3	34	10–13
G2	Null −/−	Wt +/+ (from G3)	3	25	6–11
G3	Wt +/+	Het +/− (from G2)	3	22	4–10

Pre-weaning mortality was low in all groups and unaffected by dam genotype. One wild type female had 1 pup stillborn and 2 born alive but lost between day 1 and cross fostering. Two α-casein deficient females lost 2 pups and one pup respectively between day 1 and cross fostering. No pups were lost from cross fostering until day 21, but on day 22 two pups were found dead in the largest litter (11 pups before day 22) nursed by an α-casein deficient female.

### Mouse phenotyping

All monitoring took place in a testing room adjacent to the housing room and with similar environmental conditions. The entire housing cage was moved from the housing room to the monitoring room. Physical and social aspects of mouse housing are described in detail in table 3.

**Table 3:** Details and scores of the modified SHIRPA protocol.

*Observation*	*Score*	*Definition*
Respiration rate: Observe the respiration rate in viewing jar.	0	Gasping, irregular
	1	Slow, shallow
	2	Normal
	3	Hyperventilation
Tremor: See if the mouse tremors in the viewing jar.	0	None
	1	Mild
	2	Marked
Piloerection: Observe body hair and piloerection.	0	None
	1	Coat stood on end
Palpebral Closure: Observe both eyes and their closure.	0	Eyes wide open
	1	Eyes 1/2 closed
	2	Eyes closed
Gait: Observe the gait of the mouse.	0	Normal
	1	Fluid but abnormal
	2	Limited movement only
	3	Incapacity
Pelvic Elevation: Visually measure the pelvis height of mouse	0	Markedly flattened
in gait. Keep the eye level of the observer at the height of the	1	Barely touches
mouse. Make the observation from the side of the arena.	2	3 mm elevation Normal
	3	Elevated (>3 mm elevation)
Tail Elevation: See if the mouse elevates the tail.	0	Dragging
	1	Horizontally extended
	2	Elevated/Straub Tail (>45°)
Touch Escape: Approach the mouse with a finger sideways.	0	No response
Observe how close the finger is when the mouse escapes.	1	Escape response to touch
	2	Escape response to approach
Positional Passivity: Hold up the mouse by the tail on the	0	Struggles when held by tail
arena to see if the mouse resists. If the mouse does not resist, hold the mouse in restraint by the neck, keep it on the	1	Struggles when held by neck (finger grip, not scuffed)
back, and then hold it by the hind legs. See if the mouse resists at each step and stop when the mouse resists.	2	Struggles when laid supine (on back)
	3	Struggles when held by hind legs
	4	No struggle
Trunk curl: See if the mouse brings the upper body up by stooping the ventral side and shows a sit-up movement curl	0	Absent
when held up by the tail. Twisting the upper body (Trunk sideways is not trunk curl).	1	Present
Limb grasping: See if the mouse holds the forelimbs and hind	0	Absent
legs together (Limb grasping) when held up by the tail.	1	Present
Grip Strength: Hold the mouse by the tail and drag it to the	0	None
fringe of the metal net on the arena. Evaluate the grip	1	Slight grip, semi-effective
strength felt by the hand of the observer.	2	Moderate grip, effective
	3	Active grip, effective
	4	Unusually effective
Body Tone: Pinch the mouse with the thumb and forefinger of the observer on the dorsal sides while allowing the mouse to	0	Flaccid, no return of cavity to normal
hold onto the metal net in the arena. Evaluate the resistance.	1	Slight resistance
	2	Extreme resistance, board like
Corneal Reflex: Stimulate the cornea of the mouse with the	0	None
body (not the tip) of the wire attached to a dowel and see if	1	Active single eye blink
the mouse closes the eyelids.	2	Multiple eye blink
Toe pinch: Use forceps with the tips bent to stimulate the hind	0	None
legs while allowing the mouse to hold onto the metal net on	1	Slight withdrawal
the arena. Observe the response.	2	Moderate withdrawal, not brisk
	3	Brisk, rapid withdrawal
	4	Very brisk repeated extension and flexion
Wire manoeuvre: Let the mouse hold on to a horizontal wire	0	active grip with hindlegs
with the forelimbs, hold it by the tail and bring it to horizontal position before letting go.	1	Difficulty to grasp with hindlimbs
	2	Unable to grasp with hindlimbs
	3	Unable to lift hindlegs, falls within seconds
	4	Falls immediately
Skin color: Observe the color of the ventral sides of limbs	0	Blanched
(palms and soles).	1	Pink
	2	Bright, deep red flush
	3	Dark footpad, pigmentation
Limb tone: Press the rear side of the hind legs of the mouse	0	No resistance
with the forefinger and the middle finger of the observer to	1	Slight resistance
evaluate how violently the mouse kicks back.	2	Moderate resistance
	3	Marked resistance
	4	Extreme resistance
Negative Geotaxis: Animal placed on horizontal grid, lifted to vertical with animal facing the floor - 30 s	0	animal stays in head down position
	1	animal turns head up
	2	animal turns head halfway up

### Behavioural analysis before weaning (early phenotyping)

The pups were individually weighed and monitored for general behaviour and reactivity, health, sensory and muscle development, and reflexes on day 1, 3, 7, 14 and 21 of age following a protocol based on pre-weaning development stages and adapted from [20] and [21]. Additionally, from day 13 onwards pups were weighed every second day and screened daily to detect the day when both eyes were opened. During scoring the adult female was removed from the home cage and placed into an empty opaque cage, food and water being provided. Specifically for the respective monitoring days, the following records were taken:

Day 3: presence/absence of a milk spot and the position of the animals regarding the nest (inside/outside). *Righting reflex*: the pup was positioned in dorsal recumbence and the time it took to turn to a ventral position was taken with a cut off time of 60 sec.

Day 7: presence/absence of a milk spot and the position of the animals regarding the nest (inside/outside). Presence/absence of the first fur was recorded.

Day 14: Pups were inspected for the presence of a fully developed fur and the opening of the eyelids, auditory canal and the presence/absence of the incisors. *Grip reflex*: The animals were stimulated at their 4 paws with a cotton swab and the reflex reaction (closing of paws) recorded.

Day 21: At the originally scheduled weaning day (day 21), the home cage was placed into the testing room for at least 10 minutes. After this the pups were weighed and placed individually into an empty opaque cage (observation cage; similar to the housing cage as described above) for close inspection. There each animal was screened for apparent deviations from fluid gait, horizontally extended tail position, normal body position (e.g. hunched back), normal body proportion (e.g. lumps) and normal respiration while moving freely in the cage. While being restrained in a dorsal position, signs of dehydration (lifting a skin flap between index finger and thumb), general fur condition (smoothness), the presence of wounds, trimmed whiskers, presence of discharge in nose and the anogenital region, general eye condition and skin colour were recorded. Muscle tonus in fore/hind legs and abdomen was recorded by applying slight pressure with the index finger of the free hand.

After the assessment under restraint, the following tests were performed (adapted from [20] and [21]): *Provoked biting*: To assess reflexive biting reaction, the animal was stimulated with a cotton swab at the muzzle followed by a check for conspicuous teeth and mucosa condition. *Grip strength*: To assess the ability of the animal to hold its own bodyweight, the individual was placed on a cage lid which was then turned upside down. Animals being able to hold on for at least 15 sec. were recorded as successful. *Vertical pole test*: As a test of motor coordination and balance the animal was placed on a horizontally positioned pole (80 cm long, 1.55 cm diameter, taped) which was erected slowly to vertical position. Animals falling off before reaching vertical position were recorded. *Postural reflex*: The animal was placed in an empty observation cage which was then gently shaken 3 times vertically and 3 times horizontally. The presence/absence of the natural reaction to this treatment (extending all 4 limbs) was recorded.

Thereafter, while still being in the observation cage, the senses of *touch* and of *hearing* were measured. First a sudden airflow was applied to the back of the animal from about 3 cm distance using a rubber bulb air blower and the presence of reaction (ear flick, shaking) was recorded. To test hearing, a ballpoint pen was ‘clicked’ about 5 cm behind the animal and the presence of reaction (ear flick, shaking, jumping, fleeing) was recorded. During the procedures mentioned above the number of faeces was counted. The scale pan and the observation cage were cleaned with surface cleaning wipes after each animal.

### Behavioural analysis after weaning

The animals were weighed weekly after weaning and at 8 weeks of age the animals were individually screened using a protocol adapted from [22], including parameters on health, general behaviour and reactivity, reflexes and muscle strength.

First the animal was placed into a transparent Perspex cylinder (height 18 cm, Ø 15 cm) and observed regarding general activity, respiration and occurrence of tremor. Then the animal was transferred into a Makrolon IV cage (42×30×27 cm, Allentown) and deviations from normal palpebral closure, piloerection, gait, pelvic elevation, tail elevation, and positional passivity were recorded. Touch escape response was assessed by slowly approaching the mouse with a finger sideways and attempting to stroke it.

In tail suspension, trunk curl and limb grasping were recorded with the mouse hanging and body tone, corneal/pinna/toe pinch reflex were observed while the animal was placed on a cage grid. Efficiency of grip was assessed by gently pulling the animal back by the tail. A wire manoeuvre was performed by letting the mouse hold on to a horizontal wire (length 45 cm, Ø 3.1 mm) with the forelimbs while being held by the tail and releasing it from a horizontal position.

Under supine restraint, skin colour, deviations from normal heart rate, limb/abdominal tone, lacrimation, salivation and signs of fear, irritability and aggression were recorded. Provoked biting response was tested as described above. A negative geotaxis test was performed by placing the animal on a horizontal grid and turning the grid to vertical position with the head facing down.

### Milk analysis

Milk was isolated from mammary tissue at peak lactation (day 10 of lactation) after cervical dislocation of the mice. The milk was harvested using a Pasteur pipette which was put onto the nipple while pressure was applied to the tissue. Milk was drawn up into the pipette transferred into a microcentrifuge tube and stored at −80°C.

After thawing at room temperature, milk samples were well suspended, diluted 1 in 4 in distilled water, and centrifuged for 5 sec. at 200 g in a table top centrifuge. The lower phase containing the defatted whole milk was removed from the upper lipid layer into a new tube and diluted with 2 volumes of distilled water. Then one volume of a 4× concentrated protein sample buffer was added. To separate the whey and casein fractions, defatted whole milk was spun for 15 min. at 200 g. The supernatant containing the whey fraction was diluted with 2 volumes of distilled water and one volume of a 4× concentrated protein sample buffer. The pellet containing the casein fraction was re-suspended in an equivalent of 3 volumes (of starting volume) of distilled water and then one volume of a 4× concentrated protein sample buffer was added. The samples were separated on a 10% polyacrylamide gel and stained with Coomassie Blue. Milk protein expression visible in the stained gel was then analysed on a Kodak Imaging Station using the Kodak 1D imaging software.

Total protein extracts from tissues were isolated as described [23]. Briefly tissue fragments were dounced on ice at 100 mg/ml in a buffer containing 1% SDS, 1% NP-40 and 0.5% deoxycholic acid in 1× PBS supplemented with a broad spectrum proteinase inhibitor mix (SIGMA P-8340). The lysates were incubated on ice for 30 min. and then centrifuged at 4°C for 10 min. at 10000 g. The supernatants were then aliquoted and stored at −80°C.

Cytoplasmic protein extracts for the analysis of caspase activity were prepared as described [24]. Briefly, tissue fragments were dounced on ice in a buffer containing 25 mM KPO_4_ pH 7.8, 8 mM MgCl_2_, 1 mM EDTA, 1% Triton X-100 and 15% glycerol. The extracts were incubated on ice for 5 min. and then centrifuged at 4°C for 1 min. at 10000 g. The supernatants were then aliquoted and stored at −80°C.

Western blots were done after semi-dry transfer of the proteins to a nitrocellulose membrane as described [25]. Mouse α-casein protein was detected using a rabbit-anti α-casein antiserum (Santa Cruz Biotechnology sc-98699) and a horse-radish-peroxidase linked goat anti-rabbit serum (Cell Signalling Technologies #7074). Mouse β-casein protein was detected using a goat-anti β-casein antiserum (Santa Cruz Biotechnology sc-17969) and a horse-radish-peroxidase linked rabbit anti-goat serum (Jackson Immuno-Research). Mouse grp78/BiP protein was detected using a goat-anti grp78/BiP antiserum (Santa Cruz Biotechnology sc-1051) and a horse-radish-peroxidase linked rabbit anti-goat serum. All antibodies were used at a dilution of 1∶1000.

### Mass-Spectrometry analysis

Milk proteins were separated on a 15% polyacrylamide gel and bands were excised manually. The proteins represented by these bands were trypsinised using a protocol of the Micromass MassPrep Station (Micromass Ltd, Manchester, UK) and analysed by electrospray LC-MS methods as described previously [26].

### Calcium analysis

Calcium and phosphate concentration were measured in milk derived from wild-type, α-casein deficient [−/−] and heterozygous [+/−] mice using a Konelab 30 Clinical analyser (Thermo Scientific) using the Konelab calcium (catalogue number 981367) and phosphorus (catalogue number: 981386) analysis kits as recommended by the supplier.

### RNA and quantitative PCR

RNA was isolated from tissues using the Ambion RNAwiz reagent following the supplier's protocol (1 ml of RNAwiz per 100 mg of tissue). Reverse transcription of RNA was done with MLV RNAse(-) reverse transcriptase (Promega) following the suppliers recommendations. 2 µg of total RNA was used as template for the cDNA synthesis reaction. A one in 10 dilution of the reaction was subsequently used as template for quantitative PCR (Applied Biosystems 7500 Fast System).

Quantitative PCR amplifications were done with a final primer concentration of 0.5 µM. Primer design was done using the Primer Select program of the DNA Star software suite. The sequences, annealing temperatures and amplicon sizes of the oligonucleotides used in this study are provided in Table 1. The PCR products obtained by quantitative PCR were evaluated by melting point analysis and agarose gel electrophoresis. Amplifications were done at 40 cycles of 15 sec. at 95°C, 15 sec. at the indicated annealing temperature and 30 sec. at 72°C. Data were collected at the end of each PCR cycle. Standard curves for all genes were generated from serial dilutions of a plasmid containing the cDNA for each gene. The crossing points obtained from the sample analysis was then correlated with the standard curves to provide a concentration of the individual PCR product. Expression of the casein genes was then correlated with expression of the reference genes (β-actin or GAPDH) in the same sample (expressed as pg of gene per pg of reference).

PCR arrays were carried out using a mouse apoptosis array (SABiosystems: catalogue number PAMM-012) following the recommendations of the supplier.

### Immunohistochemistry

Tissues were collected from mice at day 10 of lactation,after schedule 1 cervical dislocation. The left lower mammary gland was dissected out and placed in 4% para-formaldehyde overnight at 4°C on a rocking platform. The para-formaldehyde was then removed and the tissues were washed, first, in PBS for 30 minutes at 4°C, then in 30% ethanol for 15 min. at room temperature. This was followed by two 30 min. washes in 70% ethanol at room temperature. The tissues were then transferred to fresh 70% ethanol until ready for processing for histology. The tissues were dehydrated through a series of alcohol dilutions followed by penetration by wax under vacuum (in a Shandon Hypercentre). The tissues were then embedded in wax blocks for sectioning. Each sample was sectioned at three intervals at least 100 µm apart and a 5 µm section mounted for immuno-histochemistry analysis at each interval.

Sections were de-waxed using Histo-clear (Lamb, Inc.) for 5 min. and then re-hydrated in a graded series of ethanol (100%, 95%, 70%; 2 min. per incubation). The slides were then washed in deionised water and 1× PBS (two 5 min. incubations). The endogenous peroxidase activity was blocked by a 5 min. incubation in 3% hydrogen peroxide. After two 5 min. washes in PBS the slides were blocked in 1% BSA in 1× PBS-Tween for 20 min. After addition of the primary antibody (rabbit-anti α-casein antiserum; 1∶100 dilution in 1× PBS-Tween) the slides were incubated at 4°C overnight. The slides were then washed twice for 5 min. in 1× PBS and the secondary antibody was added (horse-radish-peroxidase linked goat anti-rabbit serum; 1∶1000 dilution in 1× PBS-Tween). After a 30 min. incubation at room temperature the slides were washed twice for 5 min. in 1×PBS-Tween.

Horseradish peroxidise activity was detected using a diaminobenzidine dye-kit (Vector-Laboratories). The slides were incubated with substrate for 5 min. The reaction was stopped by washing the slides with distilled water.

The slides were then counterstained with Haematoxylin (Cell-Path) for 2 min. and washed in running tap water. The slides were then de-hydrated in a graded series of ethanol (70%, 95%, 100%; 2 min. per incubation), cleared in Xylene (Fisher Biochemicals) and coverslips were mounted in Pertex (Cell-Path). Following staining the sections were photographed using a Leica Stereomicroscope at a 20 fold fold magnification.

### Cell culture

Mouse RAW264 cells (ECACC catalogue number 85062803) were grown in DMEM (4.5 g/l glucose) supplemented with 10% foetal calf serum, 2 mM glutamine and penecillin/streptomycin. To induce apoptosis the cells were treated with 10 µM staurosporin for 6 h. Cytoplasmic extracts for the analysis of caspase activity were prepared as described in section “milk analysis” above. Protein concentrations were measured using a Bradford assay (Sigma). Caspase activity of cell extracts was measured using the Caspase-3/7-Glo assay (Promega) as recommended by the supplier. The assay provides a pro-luminescent caspase-3/7 substrate, which contains the amino acid sequence DEVD in a reagent optimised for caspase activity, luciferase activity and cell lysis. Presence of caspase 3 or 7 in the cell extract leads to cleavage of the substrate and light emission which is detected in a luminometer (Berthold). Three or more parallel samples were analysed to measure caspase activity. 50 µl of protein extract (from cells and tissues) were mixed with 25 µl of the Caspase Glo reagent and incubated at room temperature for 20 min. after which the readings were taken. Apoptosis in RAW cells was also confirmed by measuring cell viability using the Cell-Titre Blu reagent (Promega) and DNA laddering on a 2% agarose gel.

### Data analysis

Categorical data measured only once were analyzed using the Fisher's exact test (2-sided). Count data were analyzed using the Mann Whitney U test. For comparison of body weight development we fitted a linear model for weight using time and group as covariates. Litter size was also tested as a covariate but no significant effect was observed and it was removed from the model. The interaction between time and treatment was included in the model so each treatment would have its own growth rate. We also considered a spline at day 23 (weaning threshold). The spline allows the growth rate to change after the day 23 for each treatment. This way we are able to test differences of the growth rate between treatments before and after weaning. The model was fitted using generalized estimation equations (GEE) with compound symmetry for the working correlation matrix because of the repeated measurements for weight. The comparisons of the average growth rates between the three groups were computed using the covariance matrix of the model coefficients. The data was analyzed using SPSS version 16.0 (SPSS for Windows, Release 16.0.1. 2007. Chicago: SPSS Inc.). The GEE model was fitted using R (R Development Core Team. R: A Language and Environment for Statistical Computing. Vienna, Austria. 2009). The significance level was set at 0.05.

## Results

### Generation of α-casein deficient mice

**Figure 1:**
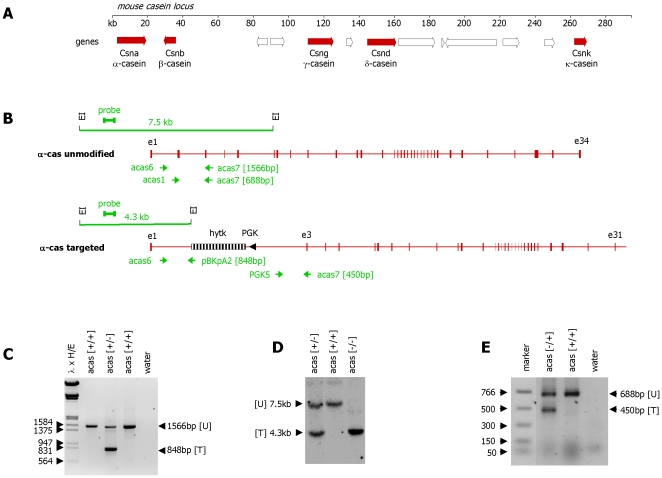
Targeting of the α-casein gene. **Panel A:** Schematic representation of the murine casein locus. Casein genes are represented as solid arrows. Other predicted genes are shown as open arrows. **Panel B:** Schematic representation of the unmodified α-casein gene and the targeted α-casein gene. Exons of the α-casein gene are indicated as solid boxes, the hytk selection marker gene is indicated as striped box. The PGK promoter element directing expression of the selection marker gene is indicated as arrowhead. The relative positions of the EcoRI restriction sites (EI), the Southern blot probe (probe), sizes of hybridising DNA fragments and the primer binding sites used for genotyping (horizontal arrows) are indicated. **Panel C:** PCR analysis of genomic DNA isolated from the three representative ES cell clones using the primer combination acas6, acas7 and pBKpA2 (analysing the 5′ end of the homologous recombination event). A 1566 bp band is detected in all samples and represents the unmodified α-casein allele [U: unmodified]. The second clone carries a targeted α-casein allele as indicated by the occurrence of a 848 bp PCR product [T: targeted]. Marker: phage λ digested with HindIII and EcoRI (λ×H/E). **Panel D:** Southern blot analysis of EcoRI digested DNA derived from tail clips of a wild-type (acas [+/+]), and α-casein mutant mice (heterozygous: acas [+/−], and homozygous α-casein [−/−]). The probe indicated in panel B detects a 7.5 kb DNA fragment representative of the unmodified α-casein allele [U] and a 4.3 kb band representative of the targeted α-casein allele [T]. **Panel E:** PCR analysis of genomic DNA isolated from two ES cell clones using the primer combination acas1, acas7 and PGK5 (analysing the 3′ end of the homologous recombination event). A 688 bp band is detected in both samples and represents the unmodified α-casein allele [U]. The second clone carries a targeted α-casein allele as indicated by the occurrence of a 450 bp PCR product [T]. Marker: NEB PCR marker.

In order to inactivate the α-casein gene (Fig. 1a) a targeting construct was established based on previous experience with constructs used to inactivate the β- and γ-casein genes [5], [27]. The targeting event removes the complete second exon which includes the translational start codon and the 15 amino acid signal peptide (Fig. 1b). In 3 independent transfections into ES cells the targeting frequency was found to be around 3%. Successfully modified ES cells clones were identified using a PCR analysing the 5′ end of the targeted gene (Fig. 1c). The targeting event was then confirmed by Southern blot analysis assessing the 5′ end of the targeted α-casein gene (Fig. 1d). The integration event was also confirmed using PCR analysis and Southern blotting with primer combinations and probes specific for the 3′ end (Fig. 1e). Two targeted cell clones were grown up and injected into blastocysts. Animals carrying a homozygous deletion of the α-casein gene are phenotypically normal prior to lactation.

### Altered milk composition in α-casein deficient mice

**Figure 2:**
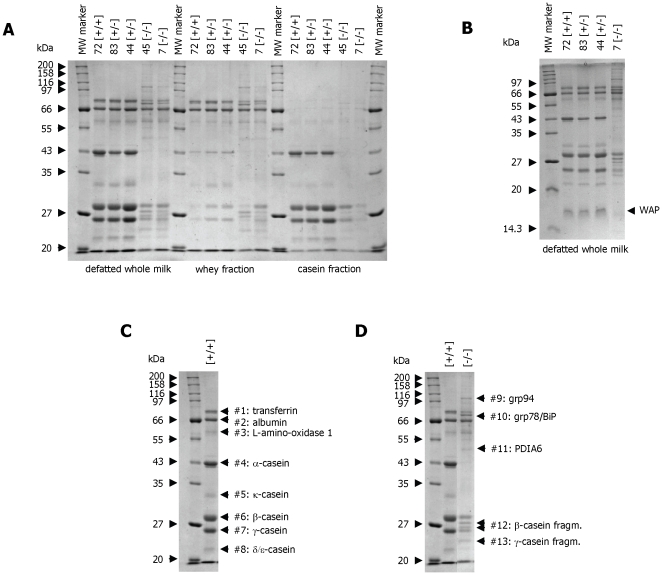
Milk protein analysis. **Panel A:** SDS-polyacrylamide gel analysis of milk derived from wild-type [+/+], heterozygous [+/−] and homozygous [−/−] α-casein deficient mice. Milk was purified as indicated in the methods section and defatted whole milk, whey and the casein fraction were separated on a 10% gel and stained with Coomassie Blue. The sizes of the protein molecular weight markers (New England Biolabs, broad range protein marker) are indicated. **Panel B:** SDS-polyacrylamide gel analysis of milk derived from wild-type [+/+], heterozygous [+/−] and homozygous [−/−] α-casein deficient mice. Defatted whole milk was separated on a 15% gel and stained with Coomassie Blue. The sizes of the protein molecular weight markers are indicated as is the position of WAP (whey acidic protein). **Panel C:** SDS-polyacrylamide gel analysis of milk derived from wild-type [+/+] mice. The proteins identified by mass-spectrometry analysis (shown in table 4) are indicated (bands #1 to 8). **Panel D:** SDS-polyacrylamide gel analysis of milk derived from wild-type [+/+] and homozygous [−/−] α-casein deficient mice. Proteins specific to milk from α-casein deficient mice identified by mass-spectrometry analysis (shown in table 4) are indicated (bands #9 to 13).

**Figure 3:**
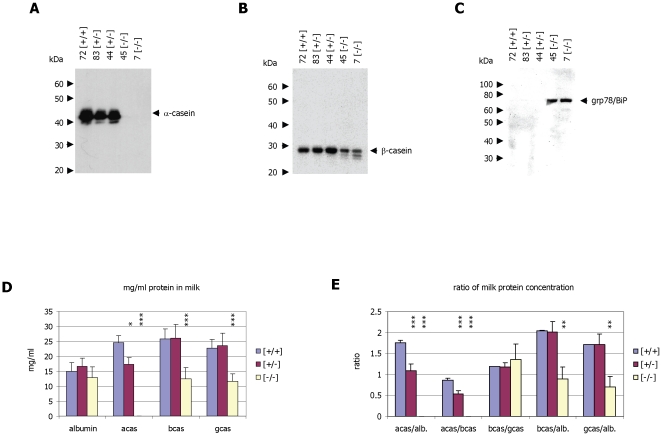
Analysis of milk protein expression. **Panel A:** Western blot analysis of milk derived from wild-type [+/+], heterozygous [+/−] and homozygous [−/−] α-casein deficient mice. The α-casein protein was detected using a rabbit-anti α-casein antiserum. **Panel B:** Western blot analysis of milk derived from wild-type [+/+], heterozygous [+/−] and homozygous [−/−] α-casein deficient mice. The β-casein protein was detected using a goat-anti β-casein antiserum. **Panel C:** Western blot analysis of milk derived from wild-type [+/+], heterozygous [+/−] and homozygous [−/−] α-casein deficient mice. The grp78/BiP protein was detected using a goat-anti grp78 antiserum. **Panel D:** Densitometric analysis of milk protein abundance as detected by SDS-PAGE. Coomassie Blue stained gels with milk samples from wild-type [+/+] (n = 3), heterozygous [+/−] (n = 6) and homozygous [−/−] α-casein deficient mice (n = 5) were scanned in a Kodak densitometer and the net intensity of the milk proteins was compared with the intensities of molecular weight marker proteins of know concentration. Protein concentrations detected in the three genotypes for albumin, α-casein (acas), β-casein (bcas) and γ-casein protein (gcas) are presented. Comparisons of [−/−] vs [+/−] as analysed by one-way ANOVA are significant with p<0.001 (***); Comparisons of [+/−] vs [+/+] are significant with p<0.05 (*) where indicated. **Panel E:** The relative amounts of milk protein abundance were calculated for the ratios of α-casein to albumin (acas/alb.), α-casein to β-casein (acas/bcas), β-casein to γ-casein (bcas/gcas), β-casein to albumin (bcas/alb.) and γ-casein to albumin (gcas/alb.). For comparisons against wild-type mice in a one-way ANOVA p<0.01 is indicated by **, p<0.001 by ***. Exact P values are presented in table 5.

Milk was isolated from lactating wild-type [+/+], heterozygous [+/−] and null [−/−] animals, separated on a denaturing protein gel and analysed by Coomassie Blue staining. Two representative samples of milk derived from heterozygous [+/−] and null [−/−] mice are shown are in Fig. 2a alongside a wild-type control milk sample. Milk from control animals displayed a series of protein bands, with the most prominent proteins corresponding to molecular weights previously observed for α-, β- and γ-casein (with apparent molecular weights of 42, 30 and 25 kDa, respectively) [28]. The identity of the protein bands was confirmed using mass-spectrometry. Table 4 details the data for the 8 most prominent protein bands indicated in Fig. 2c. These findings were also supported using Western blot analyses for α-casein and β-casein (Fig. 3a and 3b). Milk from α-casein deficient mice shows some characteristic protein bands which are absent from milk of wild-type or heterozygous mice (Fig. 2d). Mass-spectrometry analysis was used to identify some of these proteins. The major additional band with an apparent molecular weight of 78 kDa was identified as grp78/BiP. BiP is an ER resident protein which is involved in the assembly of multi-protein complexes like antibodies [29], [30]. The identity of the grp78/BiP protein was confirmed by Western blot analysis (Fig. 3c). In addition two further ER-resident proteins, grp94 and PDIA6 (protein disulfide-isomerase associated protein 6), were detected in milk of the α-casein deficient mice (Fig. 2d, table 4).

**Table 4:** Identification of milk proteins using mass-spectrometry.

*band*	*protein*	*aa*	*expected MW*	*reported MW*	*observed MW*	*MS score*	*coverage*	*number of sequences*
#1	transferrin	697	78.8 kDa		80 kDa	2040	59%	35
#2	albumin	576	67 KDa		67 kDa	1391	62%	28
#3	L-AAO-1	523	58.5 kDa		59 kDa	771	42%	16
#4	α-casein	289	33.6 kDa	43 kDa	42 kDa	1069	38%	7
#5	κ-casein	160	17.7 kDa	31 kDa	32 kDa	134	9%	2
#6	β-casein	216	23.6 kDa	26 kDa	28 kDa	512	14%	3
#7	γ-casein	169	19.5 kDa	25 kDa	26 kDa	195	27%	4
#8	δ-casein	144	15.3 kDa		22 kDa	189	46%	4
#9	grp94	802	90.1 kDa	94 kDa	100 kDa	596	37%	23
#10	grp78/BiP	656	72.5 kDa	78 kDa	76 kDa	2139	47%	29
#11	PDIA6	445	48.7 kDa		49 kDa	501	31%	10
#12	β-casein	144	23.6 kDa	26 kDa	28/27 kDa	441	12%	2
#13	γ-casein	144	15.3 kDa	25 kDa	23 kDa	435	46%	4

The number of amino acids (aa) and the expected molecular weight (MW) are given for the mature proteins (i.e. without the signal peptide) where appropriate. In addition, the reported molecular weights for the mouse caseins [28] and the molecular weights observed in the SDS-PAGE analysis are shown. The MS score is a measure of confidence of the detected protein species. A score higher than 39 is significant. The number of peptide sequences and the fraction of the total protein covered are also indicated. Protein bands #1-8 are the predominant protein species in the milk of wild-type mice. Protein bands #9-13 are detected in the milk of α-casein deficient mice but not in the milk of wild-type or heterozygous mice. L-AAO-1: L-amino acid-oxidase1, PDIA6: protein disulfide isomerase associated 6.

Milk protein abundance was then analysed quantitatively using a Kodak imaging system (Fig. 3d and e). In heterozygous mice the amount of α-casein protein is reduced by around 50% when compared to albumin (Fig. 3e). In contrast, the relative amounts of β-casein and γ-casein compared to albumin remain constant. In the milk derived from homozygous α-casein deficient mice, no α-casein protein can be detected (Fig. 2a, 3a and 3d). In addition, the amounts of β- and γ-casein are also significantly reduced (Fig. 2a, 3d and e). Whereas the ratio of β-casein to albumin in control milk is 2∶1, the ratio in milk from homozygous α-casein deficient mice is 1∶1 indicating at least a 50% reduction in β-casein protein concentration in milk (Fig. 3d and e). This reduction is also detected by Western blot analysis (Fig. 3b) Analysis of the data using one-way ANOVA demonstrated that the changes in the concentration of β- and γ-casein in the milk of α-casein deficient mice are highly significant (p>0.001; Table 5). The concentration of milk proteins (Fig. 3d) is consistent with published data [5].

**Table 5:** Significance of changes in protein ratios.

*protein ratio*	*[+/+] vs [+/−]*	*[+/−] vs [−/−]*	*[+/+] vs [−/−]*
acas/alb.	**<0.01**	**<0.001**	**<0.001**
acas/bcas	**<0.01**	**<0.001**	**<0.001**
bcas/gcas	0.0430	0.6536	0.8148
bcas/alb.	0.1676	**<0.001**	**<0.01**
gcas/alb.	0.3875	**<0.001**	**<0.01**

Milk protein expression was analysed in wild-type mice [+/+] (n = 3), heterozygous mice [+/−] (n = 6) and α-casein deficient mice [−/−] (n = 5). Expression was quantified by densitometric scanning of protein gels and correlation of the net intensities with a molecular weight marker of known concentration. The protein ratios of α-casein to albumin (acas/alb.), α-casein to β-casein (acas/bcas), β-casein to γ-casein (bcas/gcas), β-casein to albumin (bcas/alb.) and γ-casein to albumin (gcas/alb.) were determined. The data were analysed by ANOVA for one way comparisons. The p values obtained for the different comparisons are shown. P values in bold print are below the cut off points of 0.01 or 0.001 (as indicated).

In α-casein deficient milk the reduction in protein secretion also affects the mouse WAP protein, which is secreted into the whey fraction of milk (Fig. 2b). This suggests that all milk proteins secreted from mammary epithelial cells are affected by the absence of α-casein. In contrast, secretion of albumin, which is derived from serum, is not affected by the absence of α-casein (Fig. 2a). Furthermore, the overall amount of milk secreted by α-casein deficient dams appears to be reduced (observation during harvesting). Thus, α-casein deficiency causes a generalised reduction in milk secretion and a reduction in milk protein concentration.

**Figure 4:**
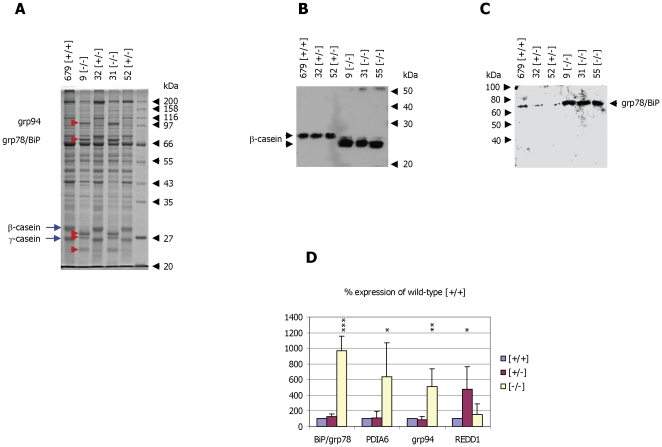
Analysis of cellular proteins in mammary tissue. **Panel A:** SDS-polyacrylamide gel analysis of total protein extracts derived from lactating mammary tissue of wild-type [+/+], heterozygous [+/−] and homozygous [−/−] α-casein deficient mice. Proteins were separated on a 10% gel and stained with Coomassie Blue. The sizes of the protein molecular weight markers (New England Biolabs, broad range protein marker) are indicated as are the positions of the β-casein and γ-casein proteins (arrows). The grp78/BiP and grp94 proteins and the breakdown products of the β-casein and γ-casein proteins are marked by arrowheads. **Panel B:** Western blot analysis of milk derived from wild-type [+/+], heterozygous [+/−] and homozygous [−/−] α-casein deficient mice. The β-casein protein was detected using a goat-anti β-casein antiserum. **Panel C:** Western blot analysis of milk derived from wild-type [+/+], heterozygous [+/−] and homozygous [−/−] α-casein deficient mice (9, 31 and 55). The grp78/BiP protein was detected using a goat-anti grp78 antiserum. **Panel D:** Correlation of gene expression in wild type [+/+], heterozygous [+/−] and α-casein deficient mice [−/−] using quantitative PCR. The results for the genes encoding the ER proteins BiP/grp78, PDIA6, grp94 and REDD1 were correlated with the expression of the reference gene β-actin. Quantification was done in 3 [+/+], 5 [+/−] and 5 [−/−] mice. Statistical analysis using one-way ANOVA demonstrates that the expression increases for BiP, grp94 and PDIA6 observed in α-casein deficient mice with respect to both wild-type and heterozygous mice occur with p<0.05. For comparisons against wild-type mice in a one-way ANOVA p<0.05 is indicated by *, p<0.01 by **, and p<0.001 by ***.

The presence of the ER proteins grp78/BiP, grp94 and PDIA6 in milk of α-casein deficient mice is unexpected. We therefore analysed whether the grp78/BiP, grp94 and PDIA6 proteins are up-regulated in lactating mammary tissue of α-casein null mice using protein gel analysis (Fig. 4a) and Western blotting (Fig. 4c). In total protein extracts derived from mammary tissue it is evident that grp78/BiP and grp94 expression is indeed up-regulated in response to the absence of α-casein. In contrast, the PDIA6 protein can not discerned in Coomassie stained gels of mammary protein extracts, as it co-localises with other proteins of similar molecular mass. Grp78, grp94 and PDIA6 are all ER resident proteins which are not normally secreted from the cells but act as chaperones, aiding protein folding. Their up-regulation is typically associated with ER stress [31], [32], [33]. This suggests that the lack of α-casein protein in mammary epithelial cells induces ER stress. Analysis of gene expression rates of grp78, grp94 and PDIA6 confirms that all three genes are significantly up-regulated in tissue samples of α-casein deficient mice with respect to control mice and heterozygous mice (Fig. 4d). In contrast, another protein often up-regulated under conditions of ER stress, REDD1 [34] is not significantly regulated by the absence of α-casein (Fig. 4d),

Several other protein bands with apparent molecular weights of 28, 27 and 22 kDa were identified in milk from α-casein deficient mice (Fig. 2d). Mass-spectrometry analysis confirmed that these proteins are breakdown products of β- and γ-casein (Table 4). The lower molecular weight β-casein products are also detected by Western blot analysis of milk samples derived from α-casein deficient mice (Fig. 3b). Analysis of protein extracts from mammary gland tissue suggests that the breakdown products are the predominant intracellular forms of β and γ-casein (Fig. 4a and b) but are not secreted as efficiently as the full length protein.

**Figure 5:**
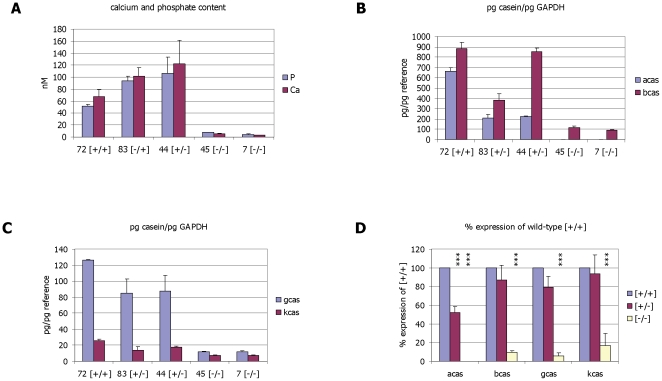
Analysis of milk calcium and phosphate levels and milk protein gene expression. **Panel A:** Calcium and phosphate content of mouse milk was determined as indicated in the methods section. Concentrations are given in nM. **Panel B:** Quantitative PCR analysis of α-casein and β-casein gene expression. cDNA derived from representative wild-type, heterozygous [+/−] and homozygous [−/−] α-casein deficient mice was analysed using primer pairs specific for α-casein, β-casein and the reference gene GAPDH. Expression of the casein genes was correlated with the reference gene and is expressed as pg casein/pg GAPDH. **Panel C:** Quantitative PCR analysis of γ-casein and κ-casein gene expression. Expression of the γ and κ-casein genes was correlated with the reference gene and is expressed as pg casein/pg GAPDH. **Panel D:** Correlation of casein gene expression in wild type [+/+], heterozygous [+/−] and α-casein deficient mice [−/−] using quantitative PCR. Casein gene expression was correlated with the expression of the reference gene β-actin. Quantification of α-casein was done in 3 [+/+], 7 [+/−] and 5 [−/−] mice. Quantification of β-casein was done in 3 [+/+], 8 [+/−] and 4 [−/−] mice. Quantification of γ- and k-casein was done in 3 [+/+], 3 [+/−] and 3 [−/−] mice. Expression in heterozygous and α-casein deficient mice is presented as percentage of median casein gene expression in wild-type control mice [+/+] (set to 100%). Error bars represent standard deviations. For comparisons against wild-type mice in a one-way ANOVA p<0.05 is indicated by *, p<0.01 by **, and p<0.001 by ***. Exact p values are presented in table 6.

The caseins are important for ion transport, mainly calcium and phosphate [35], [36]. Not surprisingly we therefore find that the lower casein protein levels in α-casein deficient mice are accompanied by a significantly lower concentration of calcium and phosphate in milk (reduced by around 90% compared to milk derived from wild-type animals; Fig. 5a).

### α-Casein deficiency affects the expression of other casein genes

We analysed the expression of casein specific mRNAs in the lactating mammary gland from control and α-casein deficient mice (Fig. 5b and c). Expression of the casein genes was correlated with expression of the reference genes GAPDH (shown in Fig. 5b and c) and β-actin (Fig. 5d). Identical results were obtained for both reference genes. As expected the amount of α-casein specific mRNA in heterozygous mice is at 50% of control levels and absent in homozygous knock-out mice. Expression of the corresponding β-casein and γ-casein casein genes is significantly reduced in α-casein deficient mice (to around 10% of the expression levels detected in wild-type and heterozygous mice; Fig. 5b, c and d). This suggests that the absence of the α-casein protein not only interferes with protein secretion from the mammary epithelial cells but that absence of both alleles of the α-casein gene also reduces the expression of the other casein genes. The expression of the κ-casein gene is also affected but not to the same extent. Its expression is reduced to around 20% of control levels in α-casein deficient mice (Fig. 5c and d). No consistent reduction of β-casein, γ-casein and κ-casein expression is detected in the heterozygous animals. Fig. 5d presents the expression of the casein genes as percentages of wild-type expression. The consistent reduction of β-, γ- and κ-casein gene expression in the α-casein deficient mammary tissue is highly significant (p<0.001; table 6) whereas expression changes in heterozygous vs wild-type mice are marginally or not significant (all p values>0.01; table 6).

**Table 6:** Significance of changes in casein gene expression.

*RNA ratio*	*[+/+] vs [+/−]*	*[+/−] vs [−/−]*	*[+/+] vs [−/−]*
α-casein	**<0.001**	**<0.001**	**<0.001**
β-casein	0.050	**<0.001**	**<0.001**
γ-casein	0.039	**<0.001**	**<0.001**
κ-casein	0.610	**<0.01**	**<0.001**

Casein gene expression was measured in wild-type mice [+/+], heterozygous mice [+/−] and α-casein deficient mice [−/−] using quantitative PCR. The results were correlated with the expression of the reference gene β-actin. Quantification of α-casein was done in 3 [+/+], 7 [+/−] and 5 [−/−] mice. Quantification of β-casein was done in 3 [+/+], 8 [+/−] and 4 [−/−] mice. Quantification of γ- and k-casein was done in 3 [+/+], 3 [+/−] and 3 [−/−] mice. The data were analysed by ANOVA for one way comparisons. The p values obtained for the different comparisons are shown. P values in bold print are below the cut off points of 0.01 or 0.001 (as indicated).

### Histological analysis of mammary tissue in α-casein deficient mice

**Figure 6:**
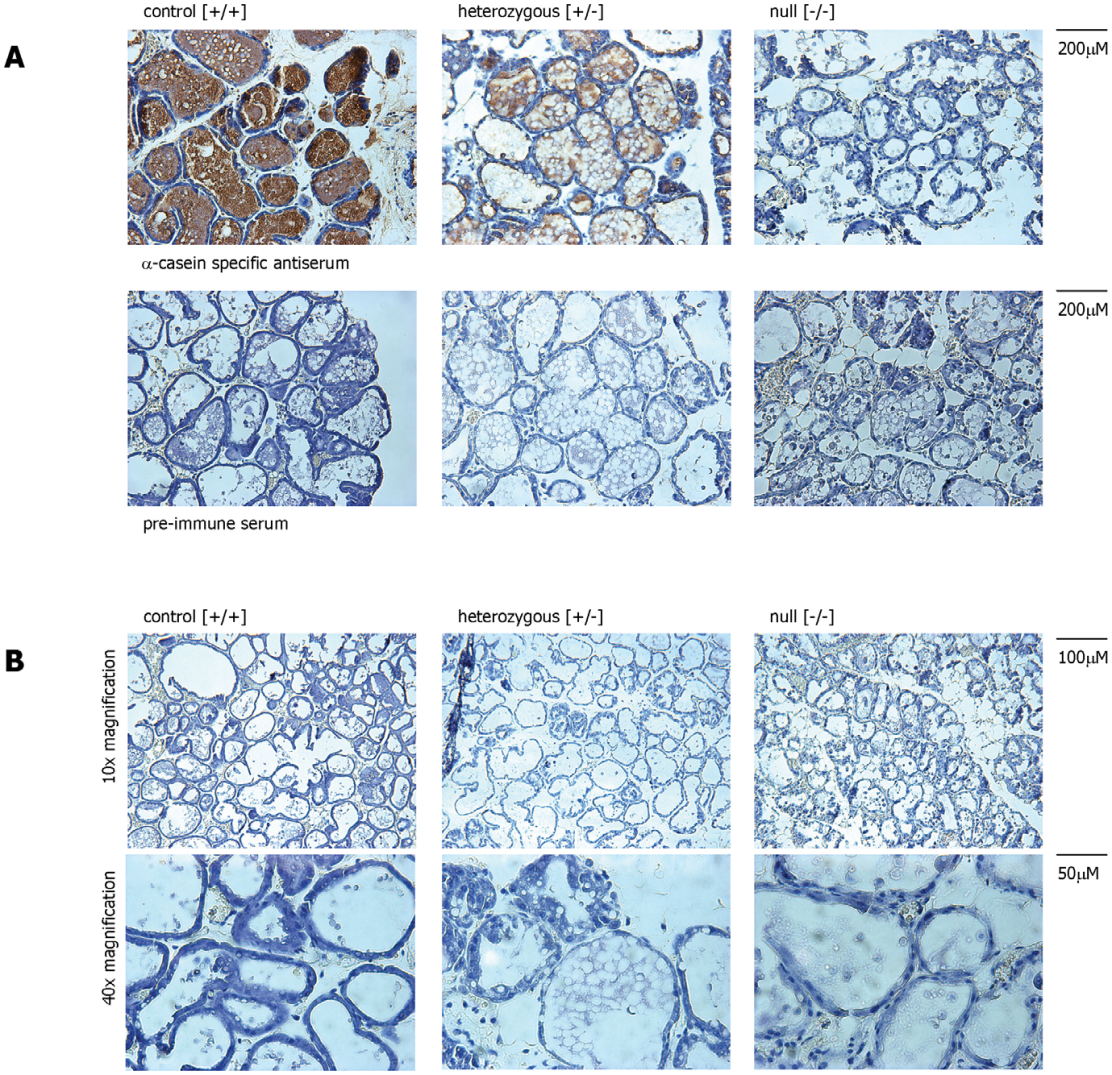
Immuno-histochemistry analysis of mammary tissue. **Panel A:** Paraffin embedded sections of mammary tissue (day 10 lactation) were analysed using a rabbit-anti α-casein antiserum. The slides were subsequently counterstained with haematoxylin. Representative sections derived from wild-type [+/+], heterozygous [+/−] and homozygous [−/−] α-casein deficient mice are presented. The lower panels are control sections incubated with a rabbit pre-immune serum in place of the α-casein specific antiserum. **Panel B:** Representative sections derived from wild-type [+/+], heterozygous [+/−] and homozygous [−/−] α-casein deficient mice at day 10 of lactation stained with haematoxylin.

**Figure 7:**
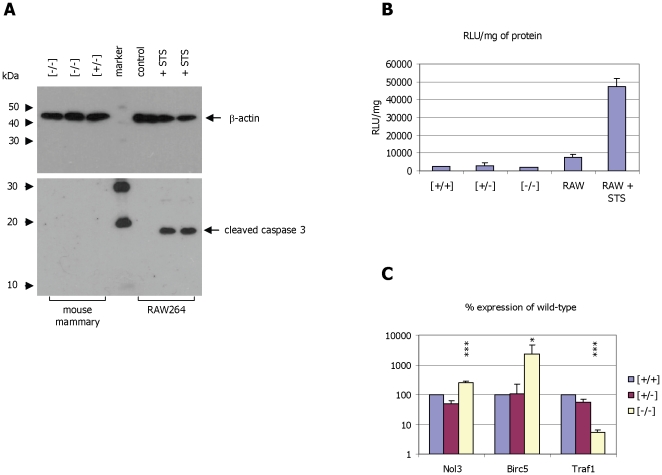
Analysis of markers of apoptosis in mammary tissue from α-casein deficient mice. **Panel A:** Western blot analysis of samples derived from two α-casein deficient mice and one heterozygous mouse (all taken at mid-lactation). The protein extracts were separated on a 10% (upper panel) and 15% (lower panel) polyacrylamide-gel blotted to nitrocellulose and detected using antisera against β-actin (upper panel) and the cleavage product of caspase 3 (lower panel). Extracts from RAW264 cells treated with 10 µM staurosporin (STS) for 6 h were used as positive control. The sizes of the protein molecular weight markers (Cell Signaling Technologies, biotinylated protein marker) are indicated as are the positions of the β-actin and caspase 3 proteins (arrows) **Panel B:** Analysis of caspase 3 and caspase 7 activity in cytoplasmic extracts of mammary gland tissue of control [+/+], heterozygous [+/−] and α-casein deficient mice [−/−] using a Caspase-Glo assay (Promega). Extracts derived from RAW264 cells treated with staurosporin were used as positive control. **Panel C:** Correlation of gene expression in wild type [+/+], heterozygous [+/−] and α-casein deficient mice [−/−] using quantitative PCR. Expression of the genes encoding the apoptosis related proteins nucleolar protein 3 (Nol3; up-regulated), Birc5 (up-regulated) and Traf1 (down-regulated) were correlated with the expression of the reference gene β-actin. Quantification was done in 3 [+/+], 6 [+/−] and 5 [−/−] mice. Statistical analysis using one-way ANOVA demonstrates that the expression changes for all three genes observed in α-casein deficient mice with respect to both wild-type and heterozygous mice occur with p<0.05. For comparisons against wild-type mice in a one-way ANOVA p<0.05 is indicated by *, and p<0.001 by ***.

In order to assess whether the reduction in protein and RNA expression is correlated with overall morphological changes, sections of mammary tissue were obtained from animals at peak lactation and analysed by haematoxylin/eosin (H&E) staining and immuno-histochemistry (Fig. 6). As expected α-casein expression was clearly detectable in tissue from wild-type control mice and heterozygous mice (Fig. 6a). In contrast no α-casein protein could be detected in sections of a-casein deficient mice (Fig. 6a). As expected sections analysed with pre-immune serum did not show any α-casein specific staining (Fig. 6a). No gross morphological alterations were detected in the sections of the different genotypes (Fig. 6b). This suggests that the absence of α-casein, although critical for overall milk composition, does not significantly impact on the survival of the mammary gland. To address this question further we measured the expression and activity of capase proteins in the mammary gland. Firstly we assessed the presence of cleaved caspase 3 in the protein extracts derived from control mice, heterozygous mice and α-casein deficient mice. No cleaved caspase 3 could be detected (Fig. 7a). In contrast cleaved caspase 3 protein with the expected molecular weight of 19 kDa was readily detected in the extracts of RAW264 cells treated with 10 µM staurosporin (Fig. 7a). Similarly, no specific signals for cleaved caspase 3 could be detected in immuno-histochemical analyses of tissue sections. In addition, caspase 3 and caspase 7 activities was measured in cytoplasmic protein extracts derived from mammary tissue of control, heterozygous and α-casein deficient mice. No significant differences in caspase 3 and 7 were detected (Fig. 7b). In contrast a significant increase in caspase activity could be detected in RAW cells treated with 10 µM staurosporin (Fig. 7b).

cDNAs derived from representative samples of control and α-casein deficient mice were analysed for characteristic gene expression changes related to apoptosis. Potential changes in candidate genes were assessed using a PCR array containing 84 apoptosis related genes. The expression of 16 genes was changed significantly (11 down-regulated and 5 up-regulated in α-casein deficient mice vs. control). Five genes, which were changed by more than 7 fold (1 down-regulated and 4 up-regulated), were further analysed in cDNAs derived from 3 control, 5 heterozygous and 5 α-casein deficient animals (Fig. 7c). Only 3 genes showed consistent and significant expression differences between control and α-casein deficient animals. Expression of the anti-apoptotic genes survivin (**b**aculoviral **i**nhibitor of apoptosis **r**epeat-**c**ontaining-5; Birc5) and nucleolar protein 3 (Nol3/ARC: nuclear **a**poptosis **r**epressor with **c**aspase recruitment domain) was increased by 22 and 2.5 fold respectively. In contrast expression of another anti-apoptotic gene, TNF receptor-associated factor 1 (Traf1), was reduced to 5% of control levels in α-casein deficient mice.

### α-Casein deficient milk restricts the growth of offspring

In order to distinguish between the effect of the genetic alteration in the offspring and the effect of the modified milk, the mice were put into 3 groups and cross-fostered such that wild-type offspring was nursed by α-casein deficient dams (3 litters with a total of 25 pups; 6 to 11 pups per litter) and heterozygous α-casein [+/−] offspring was nursed by wild-type dams (3 litters with a total of 22 pups; 4 to 10 pups per litter). Wild-type offspring nursed by wild-type dams were used as additional controls (3 litters with a total of 34 pups; 10 to 13 pups per litter; Table 2).

**Figure 8:**
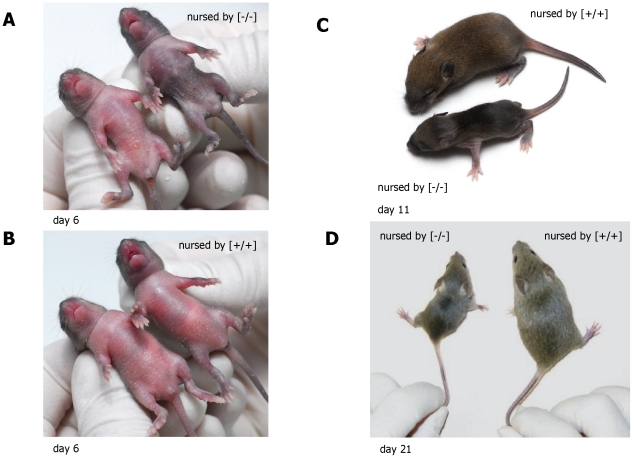
Photographs of experimental animals. **Panel A:** Photograph of wild-type mice nursed by α-casein deficient [−/−] dams (at age of 6 days). **Panel B:** Photograph of heterozygous offspring nursed by wild-type dams (at age of 6 days). **Panel C:** Photograph of mice nursed by wild-type and α-casein deficient dams at 11 days of age. **Panel D:** Photograph of mice nursed by wild-type and α-casein deficient dams at 21 days of age.

**Figure 9:**
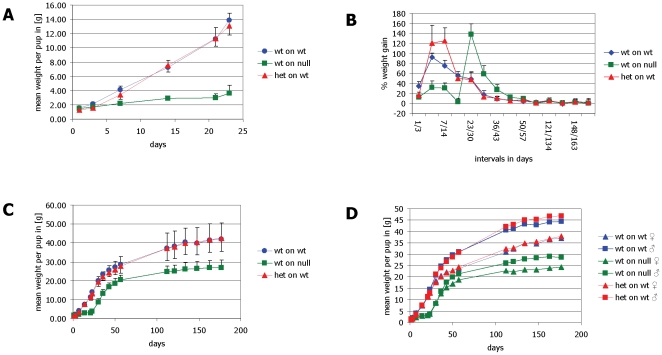
Impact of α-casein deficient milk on pup growth. **Panel A:** Growth curve of three different groups of mice during lactation (G1: wild-type pups nursed by wild-type dams n = 34; G2: wild-type pups nursed by α-casein deficient [−/−] dams, n = 25; and G3: heterozygous pups nursed by wild-type dams, n = 22). Values shown are +/− standard deviation. All weight differences between group G2 vs. G1 and G3 were significant from day 7 (p<0.001) as assessed by ANOVA. **Panel B:** Percentage weight gain throughout different stages of life for the three experimental groups. The weight of individual mice was compared on two days (as indicated: e.g. 1/3 corresponds to the interval between day 3 and day 1 of life) and the percent weight increase was recorded. The average for all mice in the three experimental groups is shown for consecutive time periods. **Panel C:** Growth curve of mice in the three groups over the first 6 months of life. Mice nursed by α-casein deficient dams show a consistent growth deficiency. **Panel D:** Growth curve of mice in the three groups of mice over the first 6 months of life separated by gender. Error bars represent standard deviations.

**Figure 10:**
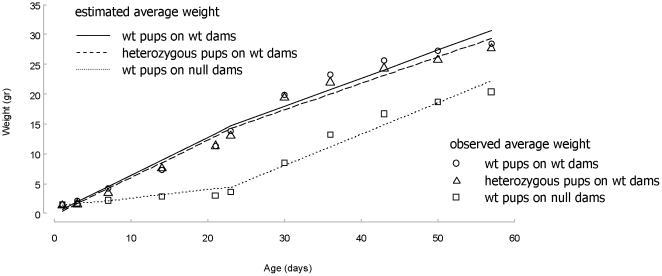
Model estimates for weight increase across experimental groups. Average growth rates were determined for wild-type pups nursed by wild-type dams (group 1), wild-type pups nursed by α-casein deficient dams (group 2) and heterozygous pups nursed by wild-type dams (group 3). The estimated increases were compared to the observed values over the first 60 days of life.

Weight gain during lactation was significantly reduced in offspring nursed by α-casein deficient dams (p<0.001 for all comparisons of offspring nursed by α-casein deficient dams with the other two groups after day 3). This effect was seen when α-casein deficient dams nursed their own pups and if the offspring of wild-type mice was nursed by deficient dams. This demonstrates that the effect is mediated by the genotype of the nursing female. This effect was also seen in two further litters after breeding of the α-casein deficient mice onto a CD1 background (Kolb et al., unpublished). All females were able to nurse their pups, as indicated by the presence of milk in the stomach (‘milk spot’) in all groups of pups (Table 3). However, by day 6, pups nursed by α-casein deficient dams were visibly emaciated (Fig. 8a) in comparison with pups nursed by control dams (Fig. 8b). By mid-lactation offspring nursed by α-casein deficient mice are significantly smaller than offspring nursed by control dams (Fig. 8c). Pre-weaning mortality was low in all groups and unaffected by dam genotype. Weighing of individual pups throughout lactation demonstrates that offspring nursed by α-casein deficient dams only reach a weight of around 3 g by the end of lactation, whereas pups nursed by control dams weigh around 12 g (Fig. 8d and Fig. 9a). After weaning mice were maintained on a standard chow diet *ad libitum*. Offspring nursed by α-casein deficient dams display a significantly accelerated growth with respect to the control group between weaning and day 50 of life (Fig. 9b and 10). However, this brief growth spurt is insufficient to bring the weight of pups nursed by α-casein deficient dams up to the weight of control animals in the long term (Fig. 9c). The difference in weight is displayed in both sexes (Fig. 9d).

Before the weaning day (day 23), wild-type pups nursed by α-casein deficient dams had a significantly slower growth rate than pups (wild-type and heterozygous) nursed by wild-type dams (p<0.001 for both comparisons). The growth rate of wild-type and heterozygous pups nursed by wild-type dams 1 and 3 was not significantly different (p = 0.729). After weaning, wild-type pups nursed by α-casein deficient dams had a significantly higher growth rate than pups (wild-type and heterozygous) nursed by wild-type dams (p = 0.039 and p = 0.014 respectively). The growth rate of wild-type and heterozygous pups nursed by wild-type dams was not significantly different (p = 0.449) For wild-type pups nursed by α-casein deficient dams the average growth rate was 0.14 g/day for days 1–23 and 0.52 g/day for days 24–60 (cf. Fig. 9b). For pups (wild-type and heterozygous) nursed by wild-type dams the growth rates were for days 1–23 0.64 g/day and 0.63 g/day, respectively, and for days 24–60 0.47 g/day and 0.45 g/day, respectively (Fig. 10).

### Physical development of mice nursed by α-casein deficient dams

In order to assess whether the deficient nutrition during lactation has an impact on general development, mice in the three experimental groups were exposed to an early phenotyping protocol including an adapted version of the general screen of the SHIRPA assay [22]. Pre-weaning observations indicated physical impairment in the ‘small’ mice. Measures of maturation (e.g. eye opening; tables 3 and 7) occur with a slight delay with respect to control animals. 60% and 50% of the pups nursed by wild-type dams had both eyes opened on day 14, all of the pups nursed by α-casein deficient females still had their eyes closed at that age, opening them only around 3 days later (Table 7). Physical abilities which rely on physical strength and coordination when tested on day 21 are significantly less developed in pups reared by α-casein deficient dams (Table 8). Significantly fewer of the growth impaired animals (36%, P-value<0.001 Fisher's exact test) were able to hold on to the pole in the vertical pole test, whereas none of the pups nursed by wild-type dams fell off before the pole reached a vertical position. Some animals nursed by α-casein deficient dams presented a rough condition of the fur (84%, P-value<0.001 Fisher's exact test) whereas all control animals had normal fur condition. Grip strength in growth impaired pups was significantly lower (P = 0.001), as 36% of the animals could not hold on to the cage lid for 15 seconds when it was turned upside down, compared to 3% and 0% in the two control groups. Animals nursed by knockout females defecated less during handling and scoring on day 21 (P-value<0.001, Mann Whitney U). Also significantly fewer (76%, P = 0.004) pups nursed by α-casein deficient females showed a provoked biting response at day 21 when compared to pups nursed by wild-type dams (100%). In summary, at pre-weaning the α-casein deficient milk suckled pups were smaller and physically weaker than those reared on wild-type milk.

**Table 7:** Eye-opening in pups.

*group (pups – dams)*	*day 14*	*day 15*	*day 16*	*day 17*	*day 18*	*median*
G1 (wt – wt)	18	14	1	0	1	day 14
G2 (wt – null)	0	0	10	14	1	day 17
G3 (het – wt)	11	11	0	0	0	day 14.5

The number of pups opening their eyes on the indicated day are presented.

**Table 8:** Behavioural differences between pups nursed by wild-type dams and pups nursed by α-casein deficient dams during the lactation period; days of assessment: 1, 3, 7, 14 and 21.

*Parameter*	*day of assessment*	*change*
milk-spot y/n	1	ns
inside/outside nest	1	p<0.05
normal/passive	1	ns
inside/outside nest	3	ns
milk-spot y/n	3	ns
normal/passive	3	ns
inside/outside nest	7	ns
milk-spot y/n	7	ns
teeth y/n	14	ns
fur y/n	14	ns
normal/passive	14	ns
grip-reflex 2/4 palms	14	ns
ears open y/n	14	ns
eyes open y/n	14	p<0.05
body-posture norm/consp	21	ns
respiration norm/consp	21	ns
dehydration n/y	21	ns
wounds n/y	21	ns
eye condition norm/consp	21	ns
whiskers trimmed n/y	21	ns
discharge from nose n/y	21	ns
condition of teeth and mucosa norm/consp	21	ns
colour of skin/ears/paws norm/consp	21	ns
tonus of abdomen norm/consp	21	ns
tonus in forelegs norm/consp	21	ns
tonus in hindlegs norm/consp	21	ns
position-reflex empty cage y/n	21	ns
sense of touch y/n	21	ns
vision y/n	21	ns
gait norm/consp	21	ns
hearing y/n	21	ns
body conformation norm/consp	21	ns
tail position norm/consp	21	p<0.05
provoked biting y/n	21	p<0.05
hanging on grid y/n	21	p<0.05
fur norm/consp	21	p<0.05
faeces weighing cage	21	p<0.05
faeces novel environment	21	p<0.05
vertical pole test	21	p<0.05

Significance level p<0.05 for Fisher's exact test. Y = yes, n = no, ns = not significant, norm = normal, consp = conspicuous.

Subsequently, post-weaning observations clearly indicated that the ‘small’ mice had, in-effect, caught up with respect to their relative physical characteristics. Specifically, when the animals were assessed again at 8 weeks of age, there were no significant differences between groups for most of the parameters observed (Table 9). This suggests that in offspring nursed by α-casein deficient mice, general development of pups is less impaired than what would be expected from their significant growth retardation which has persisted throughout their life so far (currently the mice are almost 2 years old).

**Table 9:** Comparison regarding general health and behaviour between pups nursed by wild-type dams and pups nursed by α-casein deficient dams.

*Parameter*	*P-value G1 vs G2*	*P-value G1 vs G3*	*Parameter*	*P-value G1 vs G2*	*P-value G1 vs G3*
Respiration rate	constant	constant	Irritability	0.407	constant
Piloerection	constant	constant	Fear	constant	0.407
Palpebral closure	constant	constant	Gait	>0.999	>0.999
Trunk curl	constant	constant	Grip strength	>0.999	>0.999
Limb grasping	constant	constant	Pinnareflex	>0.999	>0.999
Visual placing	constant	constant	Heartrate	>0.999	>0.999
Positional passivity	constant	constant	Limb tone	>0.999	>0.999
Aggression	constant	constant	Tail elevation	>0.999	0.514
Body tone	constant	constant	Vocalization	>0.999	0.785
Abdominal tone	constant	constant	Urination jar	0.383	0.573
Corneal reflex	constant	constant	Urination Makrolon cage	0.103	0.103
Skin colour	constant	constant	Defecation Makrolon cage	0.892	0.178
Lacrimation	constant	constant	Touch escape	0.654	0.406
Salivation	constant	constant	Toe pinch	0.084	0.623
Negative Geotaxis	constant	constant	Wire manoeuvre	0.264	0.258
Tremor	0.393	constant	Defecation jar*	0.292	0.070

P-values of parameters without significant differences between group 1 (G1: wild-type pups nursed by wild-type dams) and 2 (G2: wild-type pups nursed by α-casein deficient dams; P-value G1 vs. G2) and group 1 and 3 (G3: heterozygous pups nursed by wild-type dams; P-value G1 vs. G3) at 8 weeks of age (Fisher's exact test and *Mann-Whitney-U test). Constant values indicate that all animals in the groups compared had the same, normal, score.

## Discussion

### The role of caseins in milk protein secretion

Deficiency for α-casein severely impairs milk protein secretion in mice. In contrast β-casein deficiency does not result in a similar decline [5]. This suggests that, in contrast to β-casein, α-casein plays a more critical role in the establishment of a functional casein micelle thereby affecting secretion of all casein proteins. Alternatively or additionally due to the higher number of phosphate centres present in α-casein vs. β-casein (3 vs. 1), deficiency for α-casein (but not for β-casein) may decrease the stability of the biofluid resulting in a precipitation of calcium-phosphate in the Golgi vesicles. These results argue against a functional redundancy of the calcium-sensitive caseins (α-, β-, γ- and δ-casein). Whereas overall secretion of milk proteins is not adversely affected in heterozygous mice (apart from a 50% reduction in α-casein protein secretion), milk protein secretion from mammary epithelial cells is severely curtailed in homozygous α-casein deficient mice. This affects both, caseins and whey proteins like WAP. In contrast, secretion of albumin which is not secreted by the mammary epithelial cells is not affected by the absence of α-casein. These results suggest a functional role for α-casein in casein micelle formation and/or stabilisation (similar to that of κ-casein) [6]. We find that the glucose regulated protein 78 (grp78 or BiP) is significantly up-regulated in response to the absence of α-casein. Grp78/BiP is an endoplasmic reticulum (ER)-resident protein whose expression is enhanced under conditions of ER stress [29] [31], [33]. Surprisingly the protein is also found in milk of α-casein deficient dams. This suggests that grp78/BiP may be critically involved in the assembly of the casein micelle. One can speculate that grp78/BiP is associated with the maturing casein micelle and is co-secreted with the immature micelle into milk. Two further ER resident proteins, grp94 and PDIA6 are also secreted into milk of α-casein deficient animals indicating that the lack of α-casein has significant impacts on protein processing and transport in the mammary epithelium. Interestingly, secretion of grp78/BiP and grp94 is also observed in mice over-expressing human protein C [37], [38]. This may suggest that perturbation of the balance of milk proteins invokes a common ER stress response resulting in the co-secretion of ER resident proteins into milk. Despite the clear evidence for changes to the expression and localisation of ER resident proteins there is no clear evidence for significant increases in mammary apoptosis in the absence of α-casein. Firstly, lactation is not terminated prematurely in the deficient animals suggesting that at least a significant portion of the mammary epithelial cells is viable. Under physiological conditions mammary gland involution after weaning leads to a complete cessation of milk secretion, apoptosis and regression of the epithelial tissue [39], [40]. In homozygous protein C transgenic mice no successful lactation is established, suggesting that in that case ER stress is followed by loss of mammary epithelium function [38]. Secondly, we cannot detect any gross morphological alteration of the secretory mammary epithelium in α-casein deficient mice. Thirdly, we also failed to detect increases in caspase activity in α-casein deficient mammary tissue. Finally, the apoptosis related genes which showed consistent and significant increases in mRNA expression in mammary tissue of α-casein deficient mice include the anti-apoptotic genes survivin (Birc5) and ARC/Nol3, suggesting that the mammary cells are actively avoiding apoptosis. On the other hand a reduction in the expression of the Traf1 gene, encoding an anti-apoptotic gene in the context of the mammary gland [41] suggests a balance of pro-apoptotic and anti-apoptotic responses to α-casein deficiency. Taken together these findings may suggest that the ER-stress response in mammary epithelial cells of α-casein deficient mice is sufficient to rescue protein secretion, thereby preventing large scale apoptosis and loss of tissue function.

Interestingly, we find that steady state levels of mRNAs encoding β-casein, γ-casein and κ-casein are also severely reduced in the mammary gland of α-casein deficient mice (cf. Fig. 5d). In contrast no such reduction is observed in heterozygous mice. There could be (at least) two explanations for this. The deletion of cis-acting DNA elements in the α-casein gene may affect the expression of the entire casein gene locus. This genetic explanation would imply that the presence of one functional α-casein allele is sufficient to mediate the activation of both copies of the casein gene locus. Alternatively there may be physiological explanations. ER stress has been associated with an attenuation of protein synthesis [33]. One can speculate that in the mammary gland this leads to a decrease in the transcription of milk protein genes which account for most of the transcripts during lactation to prevent overloading of a compromised endoplasmic reticulum [33]. A decrease in the abundance of milk protein gene specific mRNAs was also observed in the transgenic mice over expressing human protein C [38]. Alternatively, the viability of the secretory mammary epithelial cells may be impaired to some degree by the ER stress response, leading to a lower relative number of casein producing cells in the mammary gland.

Not surprisingly α-casein deficiency and the reduced concentration of casein proteins is accompanied by a reduction in the concentration of both calcium and phosphate in milk. The effect of α-casein deficiency in mice is similar to that reported for a αS1-casein deficient goat breed (Cn0) in that total casein secretion is reduced by around 75% [18]. While there are no data available which suggest a critical growth delay in the offspring of αS1-Cn0 goats, it is unlikely that an allele that would have a nutritional impact similar to the inactivation of the α-casein in mice could have persisted in the natural environment. In addition, the absence of α-casein does not appear to have a critical effect on calcium secretion in goats [17]. This indicates that there may be species (and maybe also strain-) specific differences in the assembly of casein micelles.

### The consequences of α-casein deficiency for the offspring

The most critical consequence of α-casein deficiency for the offspring is a sustained shortfall in body size. Pups nursed by α-casein deficient dams only reach 25% of body weight of control animals at weaning and even at later stages in life remain around 33% lighter than control animals. The experiments described in this paper have been carried out with 3 litters of each genotype on a C57BL/6 background. However we have observed the same phenotype of reduced weight gain during lactation with two more litters on a CD1 background (Kolb et al., unpublished data), suggesting that the phenotype is similar in different genetic backgrounds. The observed changes in calcium concentration in the milk of α-casein deficient animals are unlikely to play a role in this permanent reduction in body size as calcium restriction during lactation only exerts a transient growth delay in rodents [42].

In contrast, protein deficient diets have been implicated as important regulators of metabolic programming and later health outcomes in mice and men [43], [44], [45], [46]. Gestational protein deficiency in the mother leads to intra-uterine growth delay in the offspring. However, when such offspring is then cross-fostered onto dams on a control diet, they show a phase of rapid catch up growth during lactation [47]. The combination of poor foetal and rapid post-natal growth leads to increased susceptibility for metabolic disease and reduces life-span [48], [49]. In contrast, nutritional restriction during lactation leads to a growth delay in rodent pups which is maintained throughout life [48].

Milk supply in rodents can also be modulated by a number of approaches [48], [50], [51]. Variations in litter size lead to differences in adult body weight, although generally the effects in mice are less drastic (around 10% in adulthood) than those observed in offspring nursed by α-casein deficient dams. Alternatively, milk protein concentration can be manipulated by changing the protein content of diet of the nursing dams [45]. Specific alterations in milk composition have been more difficult to achieve technically and have relied on rearing rodent pups by gastrostomal nutrition away from their mothers [52]. However, this results in a radically different early environment as these pups are not exposed to the normal maternal behaviour [53]. Therefore the effect of specific protein restrictions during lactation has not been addressed in detail.

The molecular mechanisms by which early nutrition alters body size permanently are not fully understood at the moment. But the growth hormone/IGF axis and programming of appetite control have been implicated as key mediators. Attenuated growth rates in early post-natal life provide a significant protection against metabolic disease and extend health- and lifespan [45], [51], [54], [55], [56]. Many genetic mutations of the growth hormone/IGF axis result in both, small body size and an increased life- and health-span. Increased life-span and increased resistance to metabolic disease is also observed in mice nursed by dams on a low protein diet [45]. This suggests that permanent changes in body size induced by altered protein supply during lactation are accompanied by an improvement in metabolic health. In humans formula feeding has been associated with both, a more rapid increase in body weight during lactation [57] and an increased susceptibility to metabolic disorders [46], [58], [59].

Mice reared on α-casein deficient milk show a marked delay in the development of abilities which are related to physical capabilities. However, the deficiencies displayed at weaning had disappeared at 8 weeks of age. This indicates that these impairments are transient, whereas the changes in body weight are permanent.

Thus the phenotype of α-casein deficient dams and their offspring confirm the critical role of lactation in determining life-long body size and underline the decisive role of protein supply during this developmental window. These mice represent a genetically defined model system of nutritional restriction with a significant impact on whole animal metabolism. This model can be exploited to study mechanistic aspects of metabolic programming of body size, resistance to obesity and long-term health outcomes.
